# Human teneurin-1 is a direct target of the homeobox transcription factor EMX2 at a novel alternate promoter

**DOI:** 10.1186/1471-213X-11-35

**Published:** 2011-06-08

**Authors:** Jan Beckmann, Antonio Vitobello, Jacqueline Ferralli, Daniela Kenzelmann Brož, Filippo M Rijli, Ruth Chiquet-Ehrismann

**Affiliations:** 1 Friedrich Miescher Institute for Biomedical Research, Novartis Research Foundation, Maulbeerstrasse 66, CH-4058 Basel, Switzerland; 2 University of Basel, Faculty of Science, Basel, Switzerland; 3 Current Address: Department of Radiation Oncology, Division of Radiation and Cancer Biology, Stanford University School of Medicine, Stanford, CA 94305, USA

## Abstract

**Background:**

Teneurin-1 is a member of a family of type II transmembrane proteins conserved from *C.elegans *to vertebrates. Teneurin expression in vertebrates is best studied in mouse and chicken, where the four members teneurin-1 to -4 are predominantly expressed in the developing nervous system in area specific patterns. Based on their distinct, complementary expression a possible function in the establishment of proper connectivity in the brain was postulated. However, the transcription factors contributing to these distinctive expression patterns are largely unknown. Emx2 is a homeobox transcription factor, known to be important for area specification in the developing cortex. A study of Emx2 knock-out mice suggested a role of Emx2 in regulating patterned teneurin expression.

**Results:**

5'RACE of human teneurin-1 revealed new alternative untranslated exons that are conserved in mouse and chicken. Closer analysis of the conserved region around the newly identified transcription start revealed promoter activity that was induced by EMX2. Mutation of a predicted homeobox binding site decreased the promoter activity in different reporter assays *in vitro *and *in vivo *in electroporated chick embryos. We show direct *in vivo *binding of EMX2 to the newly identified promoter element and finally confirm that the endogenous alternate transcript is specifically upregulated by EMX2.

**Conclusions:**

We found that human teneurin-1 is directly regulated by EMX2 at a newly identified and conserved promoter region upstream of the published transcription start site, establishing teneurin-1 as the first human EMX2 target gene. We identify and characterize the EMX2 dependent promoter element of human teneurin-1.

## Background

Many transmembrane proteins mediate cell-cell interactions and thereby regulate key developmental processes. Teneurins are a unique family of type II transmembrane proteins conserved from *Drosophila melanogaster *and *Caenorhabditis elegans *to vertebrates, where four paralogues exist called teneurin 1-4 [1]. This protein class was discovered in a screen for the *Drosophila *homologue of the extracellular matrix protein tenascin-C [2]. Structure and domain architecture are highly conserved across phyla. All proteins of the teneurin family share a large extracellular domain with eight tenascin-type EGF-like repeats followed by a region of conserved cysteines and YD repeats [3]. Recently, several publications suggested that the C-terminal parts of the teneurin proteins contain peptides with similarities to corticotrophin-releasing factor (CRF) and might have a function in modulating CRF-mediated behavior [4]. All vertebrate teneurins have an N-terminal intracellular domain with two polyproline motifs, EF-hand-like metal ion binding sites and several putative phosphorylation sites. This intracellular domain was shown to be cleaved from the membrane and translocates into the nucleus where it can interact with transcription factors and alter gene expression [5-7].

In *C. elegans*, RNAi knockdown and deletion of its single teneurin gene (Ten-1) results in a broad range of phenotypes, including defects in axon guidance and neuronal pathfinding, as well as gonadal disintegration and protrusion of the vulva [8-10]. *Drosophila *harbors two teneurin genes, Ten-a [2] and Ten-m/Odz [11,12]. Mutations in either of these genes result in embryonic lethality and Ten-a mutants enhance the segmentation phenotype of weak alleles of Ten-m/Odz [13]. It was also shown that teneurin expression is required for the proliferation and cellular identity in the *Drosophila *eye [14]. Extensive localization studies in mouse [15-17] and chicken [5,18-20] embryos, as well as in rat [21] and zebrafish [22] revealed that the different members of the teneurin protein family are expressed with overlapping patterns by distinct subpopulations of neurons. Experiments *in vitro *and *in vivo *showed that the different members of the teneurin family form disulfide-linked dimers [16,23] and promote homophilic cell-cell adhesions and neurite outgrowth [18,24]. These functions of the protein are believed to mediate correct pathfinding and area recognition of neurons. This was shown in the teneurin-3 knockdown mouse, which exhibits dramatic changes in the mapping of ipsilateral retinal inputs causing mismatches in binocular mapping. This is associated with major deficits in the performance of visually mediated behavioral tasks [25].

Recent findings suggest an important role for the teneurin protein family in establishing cortical arealization and patterning in the developing embryo. Teneurin-2 was found to be expressed in developing limbs, somites and craniofacial mesenchyme in a pattern strikingly similar to that of fibroblast growth factor 8 (Fgf8) and Fgf8 coated beads implanted into chicken limb buds induced ectopic teneurin-2 expression *in situ *[20]. Furthermore, teneurin-4 transcripts are down regulated, and the expression patterns of teneurins are shifted in the cortices of mice deficient in Emx2 [26]. These findings link the regulation of teneurin expression to Fgf8 and Emx2, two proteins that are part of a complex network of growth and transcription factors regulating arealization of the developing brain, a crucial event regulating sensory perception, the control of our movements and behavior (reviewed in [27]). The best studied protein in this network is Emx2. Emx2 is the vertebrate homologue of the *Drosophila *empty spiracles (*ems*) protein, which is involved in the development of the fly head [28]. This protein is a homeobox-containing transcription factor implicated in mouse cerebral cortex development [29]. It is expressed in a graded manner from rostral (low) to caudal (high) [30-33]. Knock-out and overexpression studies of Emx2 showed the function of this transcription factor in establishing the correct size and positioning of cortical areas [reviewed in 34]. Comparing expression analyses of different embryonic stages to the adult for both Emx2 [32,33] and teneurins [5,7,35] showed that areas of Emx2 expression (e.g., the cortical plate, dentate gyrus and the olfactory bulb) strongly correlate with areas of teneurin expression, suggesting a possible role of teneurins in mediating arealization.

The human teneurin-1 gene resides on the × chromosome at position Xq25, a locus with low gene density [reviewed in 36]. Beside severe mental retardation, patients suffering from a syndrome mapped to this locus also suffer from motor sensory neuropathy, deafness and severely impaired vision [37-41]. Given the predominant expression in the developing brain and its function in establishing proper connectivity in the brain, teneurin-1 is a potential target gene for causing XLMR.

In order to provide the basis for an investigation of possible deletions and mutations in teneurin-1 of XLMR patients, we decided to delineate the gene locus and determined the transcription start site(s) of human teneurin-1. We identified a novel promoter upstream of the published transcription start, which is conserved in chicken and mice. We show that EMX2 directly binds to and regulates human teneurin-1 expression at this alternate promoter.

## Results

### Identification of alternate transcription start sites of the teneurin-1 gene

**Figure 1:**
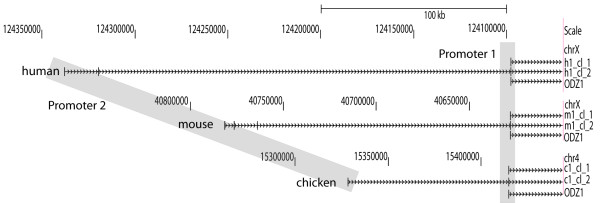
**Determination of teneurin-1 transcription start sites**. BLAT alignments to the corresponding RefSeq sequences of 2 representative clones per species obtained by 5' rapid amplification of cDNA ends are shown. The sequences of the clones are given in Table 1. The human clone h1_cl_1, the mouse clone m1_cl_1 and the chicken clone c1_cl_1 start with the annotated first exon containing the ATG translation start, whereas clones h1_cl_2, m1_cl_2 and c1_cl_2 contain up to three further non-coding exons. Based on the presence of two different transcription start sites, two alternate promoters (grey shaded areas) are postulated to control the expression of the two types of transcripts. Promoter 1 resides upstream of the first coding exon, whereas Promoter 2 is located about 100-200 kb upstream depending on the species analyzed.

Whereas the expression and localization of the different members of the teneurin protein family are well characterized, promoter regions regulating teneurin gene expression in vertebrates have not yet been studied. To find the transcription start point of human, mouse and chicken teneurin-1 (gene name is ODZ1), we performed 5'-RACE on brain cDNAs of the respective species. We used gene-specific primers derived from the first coding exon and in each case identified two classes of products (Figure 1 and Table 1). The first class ended with the 5'UTR of the published first exon containing the translation start site (as depicted in the genome browser as ODZ1), and the second class included additional non-coding exons. Two additional exons were found in human, three in mouse, and one in chicken teneurin-1. All of these exons were between 80 kb and over 200 kb distant from the first coding exon. CpG islands were found surrounding the newly identified alternate first exon suggesting promoter activity in this region. Using 4 kb of sequence surrounding the newly identified first exon of human teneurin-1 to BLAST the mouse genome revealed that this entire region was conserved between species with an overall sequence identity of 58%, and included local sequence identities of over 90%. Based on these findings, we considered that teneurin-1 expression is regulated by two different promoters that are used to differentially regulate teneurin-1 expression.

**Table 1:** Sequences obtained in 5'RACE (Translation start site in bold and underlined)

clone name	sequence
h1_cl_1	TTTTTTTTTTTTTTTGAACTGAGCTTGCTTAATCAGAG**ATG**GAGCAAACTGACTGCAAACCCTACCA GCCTCTACCAAAAGTCAAGCATGAAATGGATCTAGCTTACACCAGTTCTTCTGATGAGAGTGAAG ATGGAAGAAAACCAAGACAGTCATACAACTCCAGGGAGACCCTGCACGAGTATAACCAGGAGCT GAGGATGAATTACAATAGCCAGAGTAGAAAGAGGAAAGAAGTAGAAAAATCTACTCAAGAGAT GGAATTCTGTGAAACCTCTCACACTCTGTGCTCTGGCTACCAAACAGACATGCACAGCGTTTCTCG GCATGGCTACCATCTAGA

h1_cl_2	TTTTTTTTTTTTTTTTGGCGGGGGANCAGCACCTGGGGACGCCGCCGAAACTTGCGCTTGGAATA GGAATTACAAGGGTGACCTTTATTCCGCTGTCTCCTTTTTGATTCCCATAACTTCTGGACCTATCAA GGACTGCTTGCATTAAAGGACTTCCTCATCCTTTTTTTCATGAAACTGAGCTTGCTTAATCAGAG**A** **TG**GAGCAAACTGACTGCAAACCCTACCAGCCTCTACCAAAAGTCAAGCATGAAATGGATCTAGCT TACACCAGTTCTTCTGATGAGAGTGAAGATGGAAGAAAACCAAGACAGTCATACAACTCCAGGG AGACCCTGCACGAGTATAACCAGGAGCTGAGGATGAATTACAATAGCCAGAGTAGAAAGAGGA AAGAAGTAGAAAAATCTACTCAAGAGATGGAATTCTGTGAAACCTCTCACACTCTGTGCTCTGGC TACCAAACAGACATGCACAGCGTTTCTCGGCATGGCTACCATCTAGA

m1_cl_1	TTTTTTCCACCGCCACCTCCTCCACATGCCTGCACCTGTGCCAGGAAGCCACCTCCTACAGTGGAC TCTCTACAAAGAAGATCAATGACTACCCGCAGCCAGCCCAGCCCAGCTGCTCCTGCTCCTCCAACC AGCACACAGGATTCGGTTCATCTGCATAACAGCTGGGTCTTGAACAGTAACATACCGCTGGAGAC CAGGTACATTTTATGATTGACCATTTCAGCAAAGACTGTTTTCATTAAAGAACTTCCTTATCCTTTT TTCATGAAACTCAGCTTGCTTAATCAGAG**ATG**GAGCAAACAGACTGCAAACCTTATCAGCCTCTG TCCAAAGTCAAGCATGTCTAGA

m1_cl_2	TTTTTTCCCGCAGGAACCAGCAAAGACGCCCTAAGTCCAGCGCACTTACAGCACACCAGCAGAGC TGAGTACCTGGCAAGGAGGCGGGGGACCGCACCTGAGGACATCACTGAAACTTGCGCCTGGACT AGTCCTTCTACTGCCATGGAAACTAGATGGCACAGACAGCGGAGAGTCACTCATTCAGAACAGG GGCCCCCTTTTTAATTTCATGTCAGCCTGTTGGTCCCTGAAAGTAACTGAAAAGGAATTACAAGA GCGACTTTTATTCTGTGTAACTTCTCTTCTGGATCTAACAAGGTACATTTTATGATTGACCATTTCA GCAAAGACTGTTTTCATTAAAGAACTTCCTTATCCTTTTTTCATGAAACTCAGCTTGCTTAATCAGA G**ATG**GAGCAAACAGACTGCAAACCTTATCAGCCTCTGTCCAAAGTCAAGCATGTCTAGA

c1_cl_1	TTTTTTTCCTCATTCCTTAAGGAATTCCAGTTGCTTGTTTTCATGATTTTGAGCCTATTCAGCCAGA G**ATG**GAGCAGATGGACTGCAAACCCTACCAGCCACTGTCAAAAGTTAAACATGAAGTGGATCTA ACNTTACACAAGTTCTTCAGATGAAAGTGAAGATGGCAGAAAGCAAAGGCAATCTTATGACTCA AGAGAAACTCTGAATGAATATAGCCAAGAGCTAAGACTGAACTACAACAGTCAAGGCAGAAAAA GAAAAAATACTGACCAATCCACACAAGACATGGAATTCTGTGAGACACCCCACATTCTGTGCTCT GGCTACCAAACAGATTTACATGGTGTGTCGGAGCACAGCTACCCACTAGAGGTGGGCTCAGATG TTGATACTGAAACCGAAGGTGGCGCATCACCAGATCATGCCCTGAGGATGTGGATGAGGGGGAT GAAGTCAGAACACAGCTCCTGTCCGTCAAGCCGGGCAAACTCAGCGTTGTCCCTGACTGACACTG ACCATGAGAGGAAGTCTGATGGGGAGAATGACATGCCGGGGAGCCCACACAACCAGTTCACGTT TCTAGA

c1_cl_2	TTTTTTTCGCCGAGCCTAGAGGCGATGGGAGCTGCCGAGCCGGGGCGCTGCTGAAAGTTCAGCC GGTGGCCGCGCAGCGCGGACTCATCCTTAAGGAATTCCAGTTGCTTGTTTTCATGATTTTGAGCC CATTCAGCCAGAG**ATG**GAGCAGATGGACTGCAAACCCTACCAGCCACTGTCAAAAGTTAAACAT GAAGTGGATCTAACTTACACAAGTTCTTCAGATGAAAGTGAAGATGGCAGAAAGCAAAGGCAAT CTTATGACTCAAGAGAAACTCTGAATGAATATAGCCAAGAGCTAAGACTGAACTACAACAGTCAA AGCAGAAAAAGAAAAAATACTGACCAATCCACACAAGACATGGAATTCTGTGAGACACCCCACA TTCTGTGCTCTGGCTACCAAACAGATTTACATGGTGTGTCGGAGCACAGATACTCTAGA

### EMX2 transactivates teneurin-1 promoter reporter constructs in cell culture

**Figure 2:**
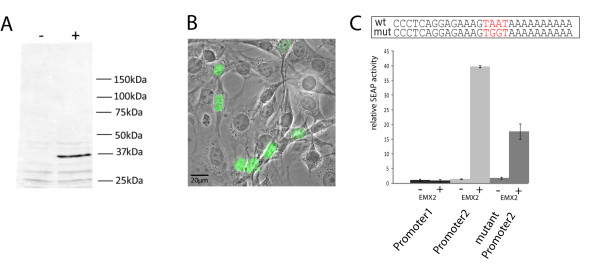
**Transcriptional activation of teneurin-1 promoter reporter constructs by EMX2**. (A) NIH3T3 cells were transfected with a flag-myc-tagged EMX2 construct or an empty vector and tested for expression by Western blot of whole cell extract with anti-Flag. (B) Immunostaining of the cells with anti-Flag reveals the nuclear accumulation of the EMX2 protein in the nuclei of the cells. (C) HEK293 cells were transfected with EMX2 or empty vector control and co-transfected with different promotor constructs as indicated. The graph shows relative SEAP values for one representative experiment performed in triplicates (n = 5). The activity obtained with the Promotor 1 construct is arbitrarily set to 1. Error bars display standard deviation of the mean. The sequences of the region harboring the putative binding site and the mutation used (mut Promoter 2) are shown in the box above the bar graph.

To test whether human teneurin-1 is a direct target gene of EMX2, we set up a reporter gene assay. We obtained a myc-flag-tagged EMX2 expression plasmid to transfect NIH3T3 cells. Recombinant EMX2 could be detected in cell extracts as a 37kD protein band on Western blots with a FLAG antibody (Figure 2a) and the protein accumulated in the nuclei of the cells as shown by immunostaining (Figure 2b). In order to do promoter reporter assays, we cloned a 4 kb fragment of highly conserved genomic sequence around the published transcription start site, as well as around the newly determined upstream transcription start site of human teneurin-1 into a pSEAP2-basic reporter vector. These promoter reporter constructs were co-transfected with the EMX2 plasmid into HEK293 cells and reporter gene activity was measured (Figure 2c). Interestingly, EMX2 was able to strongly induce reporter gene activity from the newly identified upstream promoter 2, but not from promoter 1, which remained unchanged compared to the empty vector control. Previously it was shown that EMX2 binds to a homeobox binding motif in the Wnt-1 promoter [42]. Upon sequence analysis of the promoter 2 construct we found one conserved site with a high score for EMX2 binding, while the promoter 1 construct possessed several high scoring binding sites. This indicates that the mere presence of core sequences of homeobox binding elements is not sufficient *per se *for the induction by EMX2, but the context may matter as well. To examine whether the homeobox binding motif in the promoter 2 construct contributes to the reporter gene activation upon EMX2 co-transfection, we mutated this motif and measured secreted embryonic alkaline phosphatase (SEAP) reporter gene activity. Indeed, the SEAP activity dropped significantly to 50% compared to the wild-type construct (Figure 2c).

### EMX2 transactivates a teneurin-1 promoter construct in chick embryos electroporated *in ovo*

**Figure 3:**
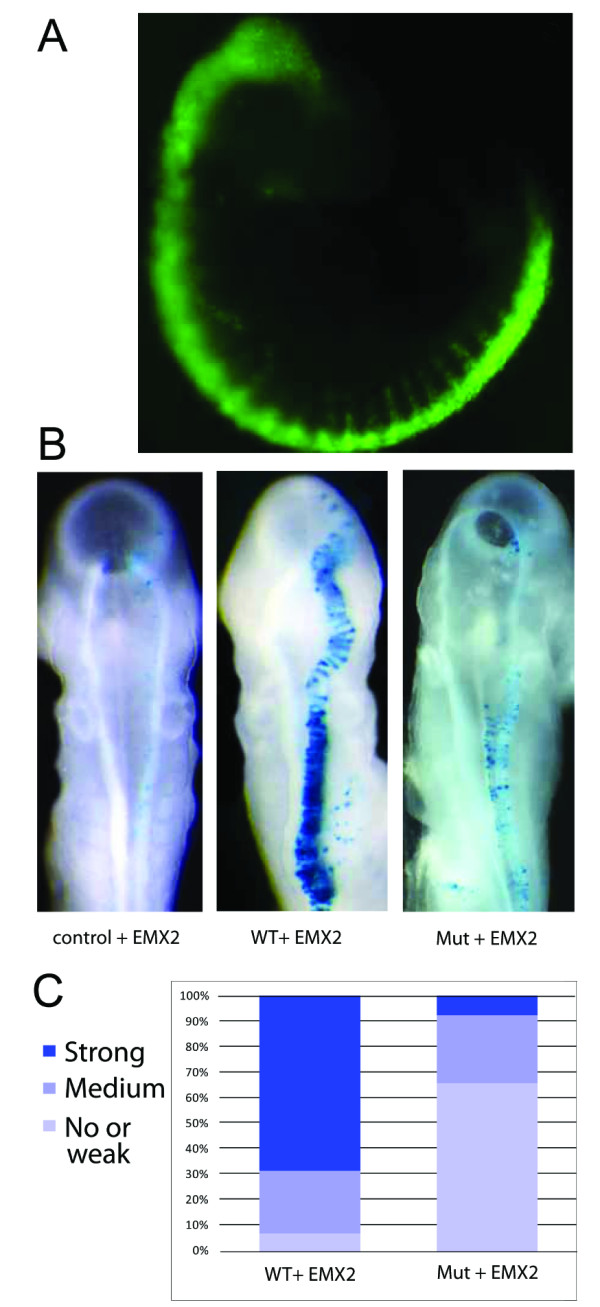
**Chicken Embryo Electroporation**. (A) Embryos were electroporated at Hamburger and Hamilton stage 10 and collected 24 h later. A CMV-GFP plasmid was always used as positive control of the electroporation and only embryos showing an expression pattern as shown were kept. Embryos electroporated with beta-globin_LacZ (BGZ40) plasmid alone or in combination with EMX2 in pCMV6-Entry (CMV-EMX2) were used as negative controls (not shown). (B) A 4 kb genomic fragment of the teneurin-1 promoter was cloned into the reporter plasmid beta-globin-LacZ (ten-1BGZ40) and electroporated alone (WT control) or in combination with CMV-EMX2 plasmid (WT + EMX2). A mutated version of ten-1BGZ40 lacking a potential EMX2 binding site (mut ten-1BGZ40) was electroporated alone (data not shown) or in combination with CMV-EMX2 (Mut + EMX2). (C) Electroporation results are summarized in stacked columns. Data are represented as percentile of the total number of electroporated embryos (n = 16 for the WT construct and n = 26 for the mutated construct) and are classified according to three levels of reporter expression: strong staining, medium staining and no or weak staining.

To further prove the promoter activity of the upstream sequence, as well as its dependence on EMX2 expression, we carried out an *in ovo *reporter gene assay in chick embryos. Upon co-electroporation of the promoter 2-lacZ construct with the EMX2 expression vector in the chick embryo neural tube, lacZ staining was strongly detected in the electroporated area (Figure 3b), whereas no staining was visible with the empty lacZ vector control (data not shown) or upon electroporation with the promoter 2-lacZ construct alone (Figure 3b). A GFP-expressing construct was always co-electroporated as well and used as a control for the efficiency of electroporation (Figure 3a).

To confirm the influence of the putative EMX2 binding motif on reporter gene activation *in vivo*, we co-electroporated the mutant promoter 2-lacZ construct with EMX2 and compared the staining with that of embryos co-electroporated with the wild-type promoter 2-lacZ construct and EMX2. Similar to the cell culture based reporter gene assay (Figure 2), reporter gene activity was also strongly reduced *in vivo*. Indeed, the vast majority of the embryos co-electroporated with EMX2 and the mutated promoter 2-lacZ construct showed a much fainter lacZ staining than those co-electroporated with EMX2 and the wild type promoter 2-lacZ construct (Figure 3b). These findings show that EMX2 is able to induce reporter gene activity at the alternate teneurin-1 promoter and that this activation is greatly dependent on an intact homeobox binding motif.

### EMX2 binds a homeobox core element in the alternate teneurin-1 promoter

**Figure 4:**
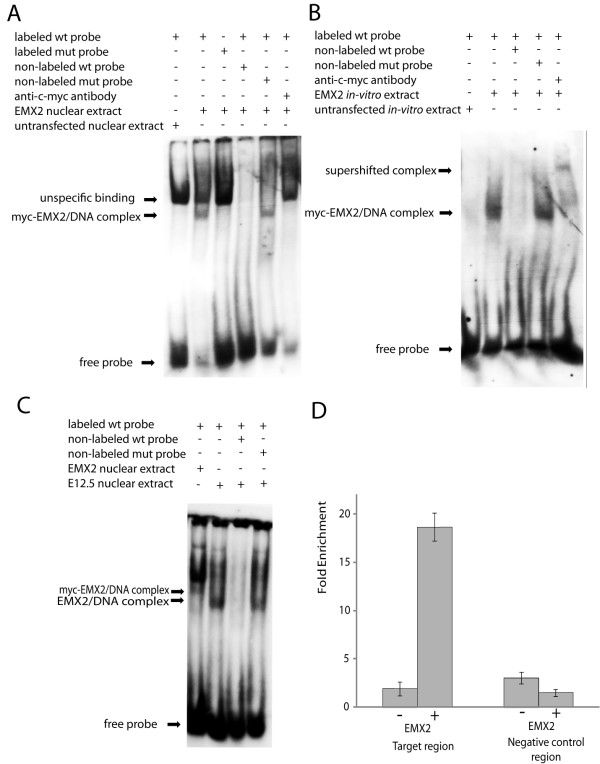
**Direct binding of EMX2 to teneurin-1 promoter oligo-nucleotide probes**. (A) Electrophoretic Mobility Shift Assays (EMSA) using EMX2 containing nuclear extract or control nuclear extract were performed in the presence or absence of Dig-labeled probe, unlabeled probe for competition, mutated probes and anti-myc antibody as indicated above the lanes. A specific myc-EMX2/DNA complex could be detected as indicated by an arrow. (B) EMSA with in vitro transcribed and translated EMX2 protein or control extracts were analyzed using Dig-labeled wildtype and mutated probes as indicated. Binding to the probe resulted in a myc-EMX2/DNA complex as indicated by an arrow that was competed by unlabeled probe and resulted in a supershifted complex after addition of anti-myc-antibody as indicated. (C) EMSAs with nuclear extracts of E12.5 embryos and nuclear extract of myc-EMX2 overexpressing cells as a control were performed using Dig-labeled wildtype probe. Binding to the probe resulting in a myc-EMX2/DNA complex and an EMX2/DNA complex is indicated by arrows. (D) ChIP of chicken embryos electroporated with flag-myc-tagged EMX2 (+) and control chicken embryos (-). Fold enrichment of the target region, containing the homeobox binding site versus a negative control region from the coding region of the same gene after anti-flag precipitation is shown. Error bars display standard deviation of the mean.

To prove that the activation of the construct is due to the direct binding of EMX2 to the homeobox binding motif in the novel upstream promoter, we performed an electrophoretic mobility shift assay (EMSA). Nuclear extracts of HEK293 cells transfected with the EMX2 construct showed a shift of the labeled probe containing the putative homeobox binding site of the upstream promoter, whereas no shift was observed in nuclear extracts of untransfected HEK293 cells or with a mutated labeled probe (Figure 4a, lanes 1-3). To show the specificity of the binding, the effect of wild-type or mutated unlabeled oligo-nucleotide on the protein/DNA interaction was analyzed. Whereas no shift of the oligo-probe was visible when competing with the unlabeled wildtype oligo-nucleotide, the shift was still detected in the presence of excess mutant unlabeled oligo-nucleotide (compare lanes 4 and 5). The complex was super-shifted with a c-myc antibody against the tagged EMX2, and indeed the shifted band disappeared (lane 6). This indicates a direct binding of EMX2 to the probe, but due to a high unspecific background the super-shifted band could not be resolved.

To reduce the unspecific background obtained with the nuclear extract, we tested EMX2 produced by *in vitro *transcription and translation in the gel shift assay. Whereas no shift of the labeled probe was detected with mock extracts, the same shifted band as with the nuclear extract could be detected with *in vitro *transcribed and translated EMX2 (Figure 4b, lanes 1 and 2), while unspecific background was greatly reduced. The binding of the protein to the probe was successfully competed with an excess of unlabeled wildtype oligo-nucleotides (Figure 4b, lane 3), whereas no competition was detected for unlabeled mutated oligo-nucleotides (Figure 4b, lane 4). Adding c-myc antibody to the binding reaction resulted in a super-shifted band (Figure 4b, lane 5), indicating a direct binding of EMX2 to the homeobox motif in the alternate teneurin-1 promoter.

To test whether an interaction between EMX2 and the binding site in the teneurin-1 promoter can also occur *in vivo *without overexpression of the EMX2 protein, we tested nuclear extracts of brains from E12.5 embryos known to express high EMX2 levels in the EMSA assay (Figure 4c). We were able to detect a shift of the band with the embryo extract, which runs lower than the complex of the overexpressed tagged protein in the control (compare Figure 4c, lanes 1, 2). We were able to compete the binding to the probe with wildtype unlabeled oligo-nucleotides, whereas no competition was detected using the unlabeled mutated oligo-nucleotide (Figure 4c, lanes 3-4). As a final proof of direct binding of EMX2 to the endogenous teneurin-1 promoter at the homeobox binding site *in vivo*, we performed chromatin immunoprecipitation (ChIP) in chicken embryos electroporated with the FLAG-myc-tagged EMX2 construct. Electroporations were performed in developing telencephalic regions in order to test the ability of Flag-myc-tagged EMX2 to bind the target region in its physiological cell context. Indeed, we detected specific enrichment of the target region containing the homeobox binding site after ChIP with the anti-FLAG antibody, recognizing the electroporated tagged EMX2 protein compared to a negative control region, which was not the case in control embryos (Figure 4d).

### Teneurin-1 expression pattern correlates with that of *EMX2 *in E14.5 embryos

**Figure 5:**
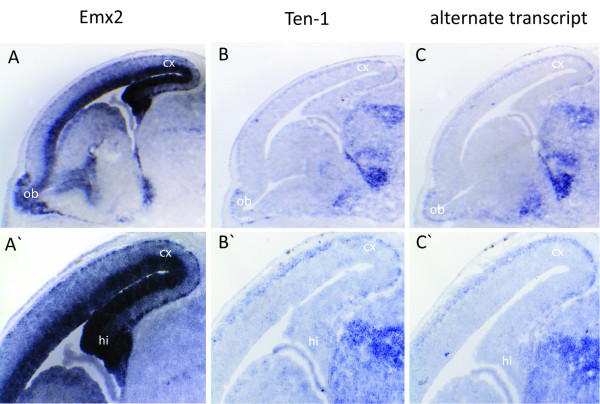
***In-situ *hybridizations for Teneurin-1 and EMX2 expression on sagittal sections at E14.5 mouse embryos**. (A; A') Expression of EMX2 in the cortex at higher magnification in A'. (B; B') Expression of total teneurin-1 in an adjacent section. (C; C') Expression of the alternate transcript of teneurin-1 in an adjacent section. Ob, olfactory bulb; hi, hippocampus

To test whether the endogenous teneurin-1 expression pattern overlaps with sites of EMX2 expression in the developing brain, we performed *in situ *hybridizations with a probe for EMX2, a probe for total teneurin-1 and an additional probe specific for the alternate transcript of teneurin-1 on adjacent sagittal brain sections (Figure 5). The staining for teneurin-1 transcripts showed expression at sites that are in accordance with those reported before for E15.5 embryos [26]. Interestingly, staining with a probe specific for the alternate transcript revealed the same staining pattern as the probe for total teneurin-1, indicating that the long transcripts are indeed expressed at these stages of embryogenesis. We detected a correlation between the teneurin-1 signals and the EMX2 signal in a caudal high to rostral low gradient. We find teneurin-1 being expressed in the marginal, but not in the ventricular zone of the cortex. Especially good correlations were found in the caudal cortex, olfactory bulb (ob) and hippocampus (hi) (Figure 5).

### EMX2 specifically induces the transcription of the alternate transcript

**Figure 6:**
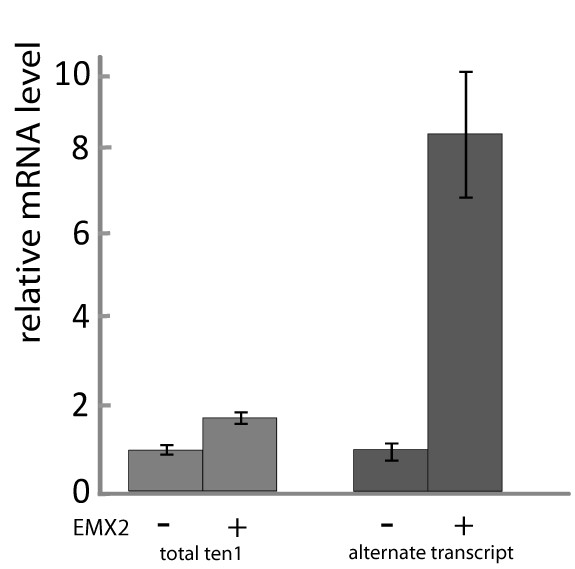
**Activation of the alternate transcript by EMX2**. RNA was isolated from parental HEK293 cells (-) and HEK293 cells stably expressing EMX2 (+). Teneurin-1 mRNA levels of 3 independent preparations were measured by real-time PCR. The graph shows total teneurin-1 mRNA (total ten1) and mRNA levels under control of the alternate promoter (alternate transcript) relative to GAPDH mRNA values. Values of the parental cells were arbitrarily set to 1. Error bars display standard error of the mean.

To test whether EMX2 is able to induce the endogenous teneurin-1 gene from the alternate promoter, we set up a real-time Q-PCR assay. We compared the mRNA expression level for total teneurin-1, as well as for the presence of the exons specific for the alternate transcript in parental HEK293-ECR cells with HEK293-ECR cells stably expressing myc-flag-tagged EMX2 (Figure 6). Indeed, the EMX2 expressing cells showed significantly (p < 0.01) elevated transcript levels of total teneurin-1. Although we generally observed a low expression level for the alternate transcript in our cells, it showed a much higher fold induction upon EMX2 expression than the total mRNA. This is further support for our reporter gene studies on the level of the endogenous gene and represents an independent confirmation that EMX2 specifically acts on the alternate promoter of teneurin-1.

## Discussion

In this work, we characterized the teneurin-1 gene locus and found novel upstream exons which are conserved between species. These new exons expand the size of the Odz1 locus to more than 800 kb, harboring one intron which is more than 200 kb in size. Genes with large introns have been reported before [43]. A continuous transcription of the entire gene, given a polymerization rate of 3800 nucleotides per minute by RNA polymerase II, would take 3.5 h [44]. This might add another level of regulation of the defined expression in time and space. Here we show that there are at least two promoters regulating teneurin-1 expression with one alternate promoter upstream of the published transcription start. Only this alternate promoter was inducible by EMX2 in reporter gene assays and cells stably overexpressing EMX2 exhibited an increase of the resulting alternate transcript. A single homeobox binding site seems to be critical for the promoter activity and is bound directly by EMX2, as shown by gel shift assay and ChIP in chicken embryos. Although one has to take into account that the teneurin-1 expression, especially in later developmental stages, opposes the expression pattern of EMX2, a direct regulation of teneurin-1 expression by EMX2 is likely to occur at earlier stages. First, we and others [26] showed that total teneurin-1 expression, as well as the expression of the alternate transcript, correlates well with EMX2 expression at E14.5. Secondly, the expression of teneurin-1 is highly dynamic and its patterned expression and the overall expression level collapses in EMX2-deficient mice [26]. Notably, we found teneurin-1 being expressed in the marginal, but not in the ventricular zone of the cortex. This suggests a possible function of EMX2 in post-mitotic neurons via the control of teneurin-1. Based on these findings, it is conceivable that the promoter at the published transcription start is responsible for the basal expression of teneurin-1, whereas the novel promoter is responsible for the graded expression dependent on EMX2. This finding suggests that this promoter region of the teneurin-1 gene is essential in establishing correct patterning of teneurin-1 expression. Although the transcription factors involved in correct patterning and arealization are well known, and their expression patterns are well characterized, very little is known about the downstream mechanisms contributing in establishing proper arealization and pathfinding [reviewed in 45]. A number of reports describe screens to find genes which are differentially expressed within the cortex [24,46,47] or which are potential target genes of differentially expressed transcription factors [11,26,48-50]. Interestingly, in both types of approaches members of the teneurin protein family were revealed as differentially expressed genes, supporting the evidence for a role of teneurin in arealization. Lists of potential target genes of the transcription factors involved in arealization, like Emx2 or Pax6, have also been described in knock-out gene expression studies [26,31,48], but indirect effects on transcription cannot be ruled out and interesting targets need to be validated. In this study, we validated teneurin-1 as the first direct target gene of EMX2 in human. As a transmembrane protein, teneurin-1 is well-suited to convey nuclear signals to the level of cell-cell interactions. However, the molecular mechanisms of how teneurins mediate their proposed function in brain development and patterning of the cortex remain to be elucidated.

Many cases of XLMR have been mapped to Xq25, the locus of the teneurin-1 gene [37-41]. Interestingly, many of these individuals suffer from motor sensory neuropathy [37], and teneurin-1 is predominantly expressed in patterns that relate to anterior sensorimotor areas [26]. Taking into account the regulation of teneurin-1 by EMX2 at the novel promoter, setting up proper arealization of the developing cortex, and the well established functions of teneurins in correct pathfinding and neurite growth, we consider teneurin-1 as a potential target gene for XLMR. When analyzing patient samples, attention should be given to the newly established promoter region, as mutations or deletions in this area could lead to a shift in expression of teneurin-1 early in the developing brain leading to improper connectivity and consequently to XLMR.

## Conclusion

In this work, we show that teneurin-1 expression is regulated by EMX2 at a novel and conserved upstream promoter. We present teneurin-1 as the first direct target gene in humans and characterize the binding site in the newly identified promoter region.

## Methods

### Rapid amplification of *5*' complementary DNA ends (5' RACE)

Total RNA of mouse and chicken brain tissue was purified with QiaShredder and RNA Easy kit (Qiagen, Hombrechtikon, CH) according to manufacturer's instructions. Using these RNA extracts and total human adult normal brain RNA (ams Biotechnology, Oxon, UK) 5'RACEs were performed with the 2nd generation 5'/3'RACE kit (Roche Diagnostic, Mannheim, Germany) according to manufacturer's instructions. Nested PCRs with the primer sequences shown in Table 2 were performed. The bands obtained were purified and cloned into vector pKS^+ ^and sequenced. The sequences were analyzed using the BLAT algorithm on human genome assembly GRCh37, mouse assembly July 2007 and chicken assembly May 2006 (http://genome.ucsc.edu) [51].

**Table 2:** Primer sequences

Primer name	Sequence
human RT-PCR	TTAGTGCATGGTCAGGTGAGG
mouse RT-PCR	TCTCCCATCTTCACTCTCATCAG
chicken RT-PCR	GCTGTGTTCTGACTTCATCC
hten PCR1 rev	GTGTCCACATCAGATCCCATCTC
hten nested **XbaI **rev	TAGT**TCTAGA**GCACAGGTGCAGGCATGAGG
mouse PCR1 rev	CTCCAGCTGGTAGCCATGTCG
mouse nested **XbaI **rev	TAGT**TCTAGA**TCTGTGTGGTAGCCGGAGCAC
chicken PCR1 rev	ATGCGCCACCTTCGGTTTCAG
chicken nested **XbaI **rev	TAGT**TCTAGA**GTAGCTGTGCTCCGACACACC
oligo dT anchor primer	GACCACGCGTATCGATGTCGACTTTTTTTTTTTTTTTT
hten1 promotor 1 **XhoI **fw	ACTA**CTCGAG**CAAGACCCATGCTGAAGCT
hten1 promotor 1 **HindIII **rev	ACTA**AAGCTT**CTCTGATTAAGCAAGCTCAGTTTC
hten1 promoter 2 **NheI **fw	ACTA**GCTAGC**CCCCTAGAGTGTTCAGCTCT
hten1 promoter 2 **EcoRI **rev	ACTA**GAATTC**GGGCCACCTCAAAAACACCTCC
mutate promoter 2 fw	CCACCCCTCACCCTCAGGAGAAAGTGGTTAAA
mutate promoter 2 rev	TTTAACCACTTTCTCCTGAGGGTGAGGGGTGG
total ten1 qPCR fw	GCATAGTTCCTGTTTGTCCA
total ten1 qPCR rev	TCTGCACATCTTGAGTAGAC
alt exon qPCR fw	GCTTGGAATAGGAATTACAAGG
alt exon qPCR rev	GAAGTCCTTTAATGCAAGCAG
hGAPDH qPCR fw	GGAGTCAACGGATTTGGTC
hGAPDH qPCR rev	AAACCATGTAGTTGAGGTC
ChIP target region fw	TTCAGCTTCCTCGTTCTTCG
ChIP target region rev	GGTGGTTACAACCGCCTTTT
ChIP negative control fw	AGATTCCTGTGAGCCCTGCT
ChIP negative control rev	TCCAACAACTCATGCAATGG

### Promoter studies

The promoter constructs for human teneurin 1 were amplified from human genomic DNA using the Expand High Fidelity system (Roche) with primer hten1 promoter 1 **XhoI **fw (all primer sequences are given in Table 2) and hten1 promoter 1 **HindIII **rev using the highlighted restriction sites for directional cloning into vector pSEAP2-Basic (Clontech, Mountain View, CA, USA) of the promoter 1 construct. For the hten1 promoter 2 construct, we used hten1 promoter 2 **NheI **fw and hten1 promoter 2 **EcoRI **rev using the highlighted restriction sites for directional cloning into the same vector. The promoter 1 construct contains a sequence of just over 2 kb from nt124097602 to nt124099666 of chromosome × and the promoter 2 construct around 4 kb from nt124336306 to nt124340205 on chromosome × of assembly GRCh37. Analysis of the promoter sequences for potential binding sites was done using the JASPAR database (http://jaspar.cgb.ki.se[52]) and the *ems *matrix. Mutation of the potential homeobox-binding sequence (nt124338584 to nt124338589 on chromosome X) in the promoter 2 was achieved using overlapping PCR with the primer set for hten1 promoter2 and mutated promoter 2 fw and rev. HEK293-EBNA cells were plated at 1 × 10^5 ^cells per well in six-well plates 18 h before transfection. Cells were transfected in DMEM containing 0.3% FCS with Fugene 6 Transfection reagent (Roche) using 1 μg promoter construct DNA and co-transfected with either empty 1 μg pcDNA3 or flag- and myc-tagged EMX2 in pCMV-Entry (OriGene, Rockville, MD, USA) as indicated in the Result section. Twenty-Four hours after transfection, the medium was collected and SEAP reporter gene activity was measured and normalized for the co-transfected plasmid pGL3, expressing firefly luciferase (Promega, Madison, WI, USA) as previously described [53].

### Real-time Q-PCR

HEK293-ECR cells were transfected as described before with the flag-myc-tagged EMX2 construct in pCMV and cells were selected for stably expressing clones with G418 (Roche) for 2 weeks. Clones were pooled and expression of the construct was tested by Western blot (data not shown). From these cells and untransfected HEK293-ECR cells RNA was isolated with QiaShredder and the RNA Easy kit (Qiagen) following the manufacturer's protocol. From this preparation, cDNA was generated using the Superscript III (Invitrogen) polymerase and random primers following the standard protocol. Real-time Q-PCR was performed on these samples with teneurin-1 specific primers and normalized to GAPDH values (sequences Table 2) using SYBR QPCR Supermix with ROX (Invitrogen) on an AbiPrism 7000 system. Three independent experiments were performed and the averaged results are shown and p-values were calculated using the one-way ANOVA.

### *In ovo *electroporation

For reporter assay experiments, chicken eggs were incubated in a humidified chamber at 38°gC and DNA constructs were injected into the lumen of the neural tube of stage Hamburger Hamilton (HH) 10-12 embryos. Construct concentrations were: 1 μg/μl lacZ reporter construct (BGZ40; [54]), 1 μg/μl EMX2 expression vector, and 0.2 μg/μl co-injected EGFP in pCMV as positive control of electroporated cells. Embryos were harvested 24 hours after electroporation and processed for β-galactosidase staining. For EMX2 overexpression, 1 μg/μl of Myc/FLAG-tagged EMX2 expression vector and 0.2 μg/μl of pCMV-EGFP construct were co-injected into the lumen of forebrain of stage HH 14 embryos. Positive tissues (n = 20 brains) were collected 72 hours after electoporation and immediately processed for chromatin cross-linking. As negative control, the same amount of unelectroporated tissue was collected and processed for ChIP experiments. Electroporations were performed as described previously using a square wave electroporator [54].

### Chromatin immunoprecipitation Assay

Brains were chopped and then cross-linked in 1% Formaldehyde (F8775, Sigma) for 10 minutes at room temperature. Cross-linking was stopped in 125 mM Glycine for 5 minutes and the material was washed three times in ice cold PBS containing EDTA-free Protease Inhibitor Cocktail (Complete, 04693132001, Roche). DNA shearing was performed in lysis buffer (50 mMTris-HCl pH8.0, 10 mM EDTA, 1%SDS, 1 × Protease Inhibitor Cocktail) using the following parameters: 20 cycles of 30 seconds ON/30 seconds OFF (Diagenode bioruptor sonicator, high power setting).

Chromatin immunoprecipitation was performed by using Dynabeads protein G (100.04D, Invitrogen) as described elsewhere [55]. The following antibodies were used: Mouse monoclonal anti-FLAG M2 (F1804, Sigma), Mouse control IgG (AB18413, Abcam).

### Electrophoretic Mobility Gel Shift Assay (EMSA)

EMX2 binding to the promoter construct was examined by Electrophoretic Mobility Gel Shift Assay (EMSA) using DIG-labeled double-stranded oligo-nucleotides (5'CAGGAGAAAGTAATTAAAAAA3' or with mutated binding site 5'CAGGAGAAAGTGGTTAAAAAA3', putative binding site underlined). For probe preparation, 5 μg of sense and anti-sense oligo-nucleotides were diluted in 90 μl TE buffer, incubated for 10 min at 95°C and cooled down for 30 min at room temperature for annealing. DIG-labeling of the probes was achieved using the DIG Gel Shift Kit, 2^nd ^Generation (Roche) according to the manufacturer's instructions. For the gel shift assay, nuclear extracts from stably EMX2 expressing HEK293 cells, *in-vitro *translated extracts or nuclear extracts of E12.5 embryo brains containing 20 μg of total protein were incubated with 4 μl of 5 × binding buffer of the Gel Shift Kit, 1 μg double-stranded poly(dIdC) and 0.1 μg poly-L-lysine in a 19 μl reaction mix. For the competition assay, unlabeled wild-type or mutant annealed oligo-nucleotide were added with a 150-fold excess. This mix was incubated for 20 min at room temperature. Afterwards, 1 μl of labeled probe was added and the mix was incubated for another 20 min at 30°C. For supershifts, 1 μl of c-myc antibody (Sigma) was added after 10 min incubation with the labeled probe. Following another 10 min of incubation, the reaction mix was loaded onto a precast 6% DNA retardation gel (Invitrogen, Carlsbad, CA, USA), which was pre-run in 0.5 × TBE for 20 min at 80 V and 4°C. The gel was run for 1.5 h at 80 V and 4°C. After separation, the complexes were blotted on a positively charged nylon membrane in 0.5 × TBE for 45 min at 280 mA and DIG detection was performed as described in the manufacturer's instructions.

### *In-vitro *transcription and translation of EMX2

*In-vitro *transcription and translation of EMX2 was achieved using the TNT^® ^Coupled Reticulocyte Lysate System (Promega). 25 μl of TNT^® ^rabbit reticulocyte lysate, 2 μl TNT^® ^reaction buffer, 1 μl TNT^® ^T7 RNA polymerase, 0.5 μl of each Amino Acid mixture without Leucine and without Methionine and 1 μg of EMX2-pCMV-Entry were mixed in a 50 μl reaction mix and incubated for 90 min at 30°C, quick frozen in dry ice/ethanol and stored at -80°C until used in EMSA.

### *In-situ *hybridization

*In-situ *hybridizations on sections were performed as previously described [56]. The following RNA probes were used: For EMX2 we used the entire CDS of EMX2 (NM_010132.2), for total teneurin-1 we used the probe previously published [26] and for the probe specific for the alternate transcript we used the sequence described in Table 1 (m1_cl_2) plus the first 100 bp of the CDS of mouse teneurin-1 (NM_011855.3).

## Authors' contributions

JB prepared all constructs, participated in the 5'RACE, performed sequence alignments, EMSA and QPCR and prepared the first draft of the manuscript. AV performed the chicken electroporation and the ChIP experiments as well as the mouse *in-situ *hybridization. JF performed the SEAP assay and validated EMX2 expression. DKB performed the 5'RACE in chicken and mouse. FMR participated in the planning and discussion of the experiments. RCE participated in the planning and discussion of the experiments, writing the paper and preparation of the figures. All authors read and approved the final manuscript.

## Abbreviations

XLMR: X-linked mental retardation; hten1: human teneurin-1; EMSA: Electrophoretic Mobility Shift Assays; RACE: rapid amplification of cDNA ends; CRF: corticotrophin-releasing factor; SEAP: secreted embryonic alkaline phosphatase; ChIP: Chromatin Immunoprecipitation
